# Optimal duration of antimicrobial prophylaxis in patients undergoing distal pancreatectomy: A multicenter cohort study

**DOI:** 10.1002/ags3.12903

**Published:** 2025-01-02

**Authors:** Kenjiro Okada, Kenichiro Uemura, Sohei Satoi, Tsutomu Fujii, Manabu Kawai, So Yamaki, Toru Watanabe, Hideki Motobayashi, Shinya Takahashi

**Affiliations:** ^1^ Department of Surgery, Graduate School of Biomedical and Health Sciences Hiroshima University Hiroshima Japan; ^2^ Department of Surgery Kansai Medical University Osaka Japan; ^3^ Department of Surgery and Science, Faculty of Medicine, Academic Assembly University of Toyama Toyama Japan; ^4^ Second Department of Surgery, School of Medicine Wakayama Medical University Wakayama Japan

**Keywords:** anti‐infective agents, pancreatectomy, pancreatic fistula, risk factors, surgical wound infection

## Abstract

**Background:**

Antimicrobial prophylaxis is routinely administered in patients undergoing distal pancreatectomy, with cephalosporins being the most frequently used agents. However, there is limited evidence regarding optimal duration of antimicrobial prophylaxis. This study aimed to evaluate the optimal duration of antimicrobial prophylaxis in distal pancreatectomy.

**Methods:**

A multicenter cohort study was performed using a common database of patients who underwent distal pancreatectomy between April 2017 and March 2022 at four high‐volume centers in Japan. Eligible patients were divided into two groups according to the duration of antimicrobial prophylaxis: intraoperative or up to 24 h after surgery and more than 24 h after surgery. Primary endpoint was the incidence of surgical site infections.

**Results:**

A total of 496 patients were enrolled in this study, including 254 and 242 patients categorized into the intraoperative or up to 24‐h and more than 24‐h groups, respectively. Surgical site infections occurred in 129 patients (26%). The intraoperative or up to 24‐h group had a significantly lower incidence of surgical site infection (19% vs. 33%, *p* < 0.001) and infectious clinically relevant postoperative pancreatic fistula (8% vs. 17%, *p* = 0.002). There were no significant differences in severe surgical site infection rates between the groups. Multivariate logistic regression identified more than 24‐h administration of antimicrobial prophylaxis as an independent risk factor for surgical site infections (*p* = 0.001).

**Conclusion:**

Prolonged administration of antimicrobial prophylaxis may not be effective in preventing surgical site infections after distal pancreatectomy compared to intraoperative or up to 24‐h administration.

## Graphical abstract

Prolonged administration of antimicrobial prophylaxis might not be effective to prevent surgical site infections after distal pancreatectomy compared to intraoperative or up to 24‐h administration.

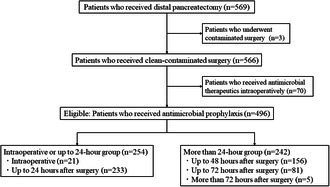

## 1 INTRODUCTION

Antimicrobial prophylaxis is essential for the management of postoperative infections in gastrointestinal surgery, which is associated with a higher incidence of surgical site infections than other surgeries. The appropriate use of antimicrobial prophylaxis is important, as prolonged administration of antimicrobial prophylaxis can result in antimicrobial resistance or microbial substitution. The World Health Organization guidelines^1^ or American clinical practice guidelines^2^ for preventing surgical site infections recommend that antimicrobial prophylaxis be administered intraoperatively or up to 24 h after surgery, with cephalosporins being the most frequently used agents for gastrointestinal procedures. Several randomized clinical trials of antimicrobial prophylaxis for gastrectomy^3^, ^4^, ^5^ and colectomy^6^ have been performed. With regards to elective gastrectomy for gastric cancer, intraoperative administration of antimicrobial prophylaxis did not increase the incidence of surgical site infections compared to postoperative administration and is therefore recommended for intraoperative use only. However, with respect to elective colectomy for colorectal cancer, it is unclear whether there is a difference in the usefulness of intraoperative administration alone and postoperative administration. Furthermore, there is little evidence regarding the optimal duration of antimicrobial prophylaxis in gastrointestinal surgical procedures other than gastrectomy and colorectal resection (i.e., esophageal surgery, hepatobiliary, and pancreatic surgery).^7^

Pancreatic surgery has a high risk of surgical site infection, and antimicrobial prophylaxis is widely used for varying durations postoperatively. Pancreatoduodenectomy includes pancreatic anastomosis and gastrointestinal reconstruction. The incidence of postoperative infectious complications, including postoperative pancreatic fistula (POPF), is relatively higher than that for other gastrointestinal surgeries.^8^, ^9^ A randomized clinical trial^10^ reported that single‐day prophylactic use was appropriate for pancreatoduodenectomy following biliary drainage without cholangitis, which can increase the risk of postoperative infectious complications.^11^ In distal pancreatectomy, the incidence of postoperative complications, including POPF, remains high at 10–30%.^12^, ^13^ However, distal pancreatectomy does not involve gastrointestinal anastomosis, and the incidence of postoperative infections could be relatively lower than that after pancreatoduodenectomy. From the viewpoint of upper abdominal surgery, gastrectomy may be comparable to distal pancreatectomy, and evidence from randomized controlled trials^3^, ^4^, ^5^ suggests that intraoperative or up to 24‐h antimicrobial prophylaxis may be appropriate for distal pancreatectomy. Nevertheless, the types of postoperative complications differ significantly between gastrectomy and distal pancreatectomy. As a result, the optimal duration of antimicrobial prophylaxis for distal pancreatectomy remains unclear. Therefore, this large‐scale multicenter cohort study was conducted to evaluate the optimal duration of antimicrobial prophylaxis in distal pancreatectomy.

## 2 METHODS

### 2.1 Study design

This multicenter cohort study included patients who underwent distal pancreatectomy for pancreatic disease between April 2017 and March 2022 at four high‐volume surgical institutions in Japan: Kansai Medical University Hospital, Toyama University Hospital, Wakayama Medical University Hospital, and Hiroshima University Hospital. First generation cephalosporins were generally administrated as perioperative antimicrobial prophylactic agents in this study, and second generation cephalosporins were also administrated when first generation cephalosporins could not be used during the study period. Patients who underwent contaminated surgery were excluded from this study. Patients who received antimicrobial therapeutics such as third/fourth generation cephalosporins or carbapenem intraoperatively were also excluded. Those without preoperative infections were eligible for inclusion in this study. Patients were categorized into two groups based on the duration of antimicrobial prophylaxis: intraoperative or up to 24 h after surgery and more than 24 h after surgery. When postoperative infection occurred clinically and the patient was switched from antimicrobial prophylaxis to antimicrobial therapeutics, the duration of antimicrobial therapy was not included. The primary endpoint of this study was the incidence of surgical site infection within 30 days of surgery. The secondary endpoints were the incidence of remote infection, clinically relevant postoperative pancreatic fistula (CR‐POPF), postoperative complications other than infectious complications, length of postoperative hospital stay, readmission, and 90‐day mortality. This study was approved by the Institutional Review Board of Hiroshima University and was conducted in accordance with the principles of the Declaration of Helsinki (approval number: E‐2022‐0227).

### 2.2 Postoperative infection

Postoperative infections included surgical site and remote infections. Surgical site infections were categorized as superficial, deep incisional, or organ/space infections. Remote infections were classified as respiratory tract infection, antibiotic‐associated diarrhea, urinary tract infection, catheter‐related bloodstream infection, drain infection, or bacteremia of unknown origin, in accordance with the National Healthcare Safety Network infection criteria.^14^ The presence of POPF was determined according to the International Study Group of Pancreatic Fistula criteria.^15^ A single closed drain was routinely placed near the pancreatic stump remnant. Grade B and C POPF were defined as CR‐POPF and classified into infectious and non‐infectious CR‐POPF. The incidence of postoperative infection was monitored for 30 days postoperatively, and the severity was graded according to the Clavien–Dindo classification system.^16^ Grades III, IV, and V were defined as severe. Readmission was defined as admission within 30 days of discharge from the initial surgery. Mortality was defined as surgery‐related death occurring within 90 days of surgery.

### 2.3 Data collection

Patient characteristics, including age, sex, body mass index (BMI), American Society of Anesthesiologists physical status classification (ASA‐PS), combined cardiovascular and pulmonary disease, diabetes, laparotomy, smoking, steroid use, prognostic nutritional index (PNI), pancreatic disease, and whether or not neoadjuvant therapy was administered, were collected. Surgical data included antimicrobial prophylaxis duration, surgical procedure, texture of pancreas, thickness and method of pancreas transection, combined vascular and organ resection, operation time, estimated blood loss, and transfusion. The postoperative outcome data included surgical site and remote infections, CR‐POPF, non‐infectious postoperative complications, postoperative hospital stay, readmission, and 90‐day mortality.

### 2.4 Statistical analysis

Continuous variables are presented as median and interquartile range (IQR). Categorical variables were compared using the chi‐squared test or Fisher's exact test, while continuous variables were compared using the Mann–Whitney U test. Propensity score matching (PSM) was performed to adjust for selection bias. A propensity score for each patient was estimated using a logistic regression model, with duration of antimicrobial prophylaxis as the dependent variable. Covariates included clinicopathological factors potentially associated with postoperative infections. After calculating the propensity scores, 1:1 nearest neighbor matching was applied with a caliper of 0.2. Risk factors were evaluated using univariate and multivariate logistic regression models, and odds ratios (OR) and 95% confidence intervals were reported. All tests were two‐sided, and *p* < 0.05 was considered significant. Statistical analyses were performed using the JMP software (version 17.0; SAS Institute, Cary, NC, USA).

## 3 RESULTS

### 3.1 Participants

**FIGURE 1:**
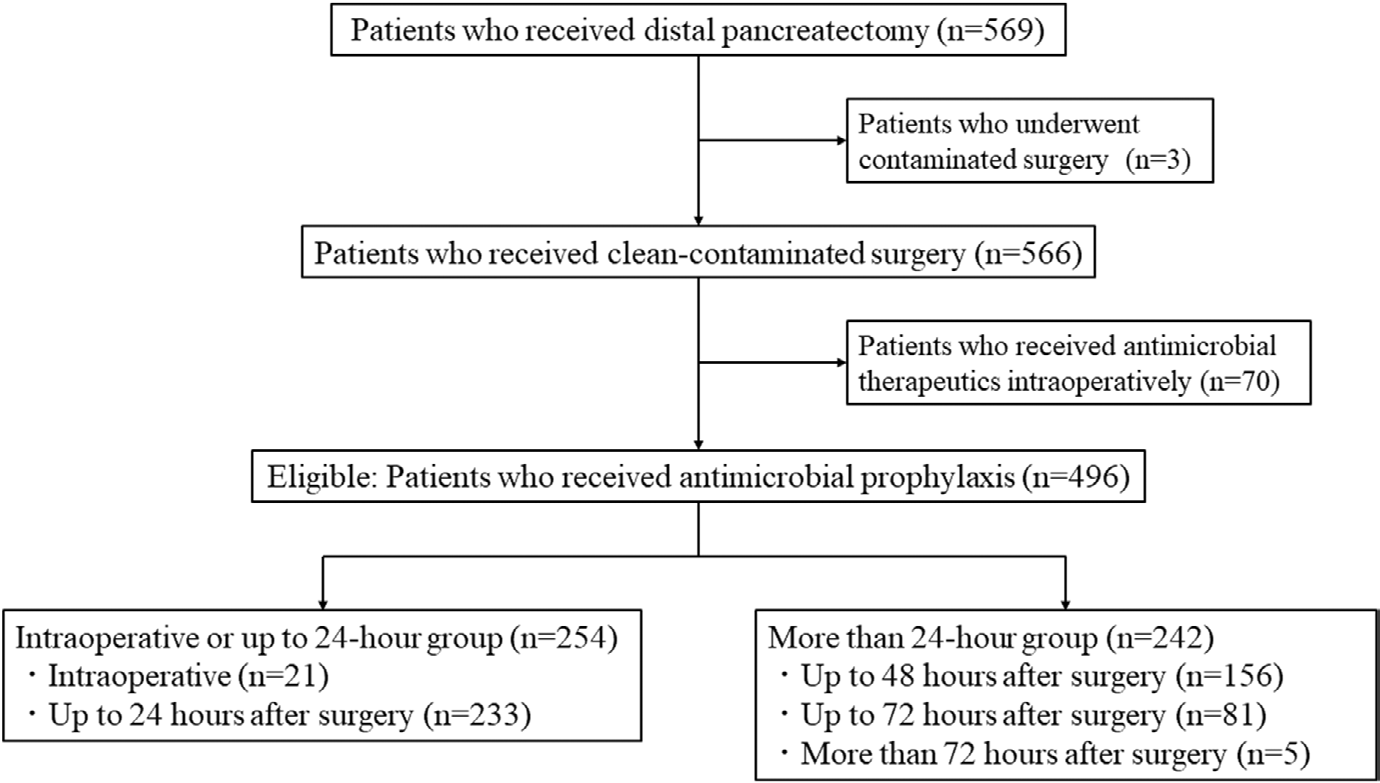
Patient flow diagram of this study.

This study enrolled 569 consecutive patients who underwent distal pancreatectomy at four high‐volume Japanese centers between April 2017 and March 2022. Of these patients, three who underwent contaminated surgery and 70 who received antimicrobial therapeutics such as third/fourth generation cephalosporins or carbapenem intraoperatively were excluded. The remaining 496 patients were eligible for this study. The enrolled patients were divided into the intraoperative or up to 24‐h administration of antimicrobial prophylaxis (*n* = 254) and more than 24‐h groups (*n* = 242), respectively. The intraoperative or up to 24‐h group included those who received antimicrobial prophylaxis intraoperatively (*n* = 21) and up to 24 h after surgery (*n* = 233). The more than 24‐h group included those who received antimicrobial prophylaxis for up to 48 h after surgery (*n* = 156), up to 72 h after surgery (*n* = 81), and more than 72 h after surgery (*n* = 5). The patient flow diagram is summarized in Figure 1.

Surgical site infections occurred in 129 patients (26%), including 15 superficial incisional surgical site infections (3%), four deep incisional surgical site infections (1%), and 113 organ/space surgical site infections (23%). CR‐POPF occurred in 80 patients (16%), including 61 infectious POPF (12%) and 19 non‐infectious POPF (4%). Severe surgical site infections were observed in 44 patients (9%). Remote infections occurred in 10 patients (2%), including five cases of respiratory tract infections, two urinary tract infections, one antibiotic‐associated diarrhea, and others. Severe remote infections were observed in three patients (1%). Antimicrobial therapy was administered to 127 patients (26%) with postoperative infections. Non‐infectious postoperative complications occurred in 91 patients (18%). The median postoperative hospital stay was 14 days (IQR, 10–22 years). Readmission was required for 25 patients (5%), and 90‐day mortality occurred in one patient (0.2%).

### 3.2 Comparisons between the intraoperative or up to 24‐h and more than 24‐h groups

Comparisons of patient demographics and postoperative outcomes between the two groups are summarized in Table 1. In terms of baseline characteristics, the intraoperative or up to 24‐h group had a significantly lower incidence of ASA‐PS ≥3, cardiovascular diseases, pulmonary diseases, and smoking, whereas they had a significantly higher rate of malignant tumors. Other characteristics, including age, sex, BMI, diabetes, laparotomy, steroid use, PNI and neoadjuvant therapy, were not significantly different between the two groups. With regards to surgical factors, the intraoperative or up to 24‐h group had a significantly lower incidence of soft pancreas, stapler transection, and longer operation time. Other surgical factors, including procedures, thickness of pancreas transection, combined vascular and organ resection, estimated blood loss and transfusion, were not significantly different between the two groups.

**TABLE 1:** Comparisons of patient characteristics and surgical outcomes between the intraoperative or up to 24‐h and more than 24‐h administration groups (*n* = 496).

Factors	Antimicrobial prophylaxis		*p*
	Intraoperative or up to 24‐h, *n* = 254	More than 24‐h, *n* = 242	
Patient demographics			
Age, ≥80 years	42 (17)	33 (14)	0.367
Sex, female	140 (55)	118 (49)	0.157
BMI, ≥22 kg/m^2^	120 (47)	118 (49)	0.736
ASA‐PS, ≥3	20 (8)	35 (14)	0.019
Cardiovascular disease	25 (10)	39 (16)	0.037
Pulmonary disease	14 (6)	42 (17)	<0.001
Diabetes	74 (29)	86 (36)	0.127
Laparotomy	70 (28)	85 (35)	0.069
Smoking	70 (28)	89 (37)	0.028
Steroids	5 (2)	3 (1)	0.517
PNI, <45	59 (23)	64 (26)	0.407
Pancreatic disease, malignant	161 (63)	120 (50)	0.002
Neoadjuvant therapy	80 (32)	81 (33)	0.639
Procedures, minimally invasive	108 (43)	102 (42)	0.933
Texture of pancreas, soft	202 (80)	221 (91)	<0.001
Thickness of pancreas transection, ≥15 mm	79 (31)	79 (33)	0.713
Method of pancreas transection, stapler	221 (87)	226 (93)	0.016
Vascular resection	22 (9)	25 (10)	0.526
Organ resection	26 (10)	22 (9)	0.666
Operation time, ≥300 min	87 (34)	118 (49)	0.001
Estimated blood loss, ≥400 mL	65 (26)	73 (30)	0.256
Transfusion	14 (6)	17 (7)	0.486
Postoperative outcomes			
Surgical site infections	49 (19)	80 (33)	<0.001
Superficial	5 (2)	10 (4)	0.156
Deep	2 (1)	2 (1)	0.961
Organ/space	43 (17)	70 (29)	0.001
Infectious CR‐POPF	20 (8)	41 (17)	0.002
CD ≥IIIa	24 (9)	20 (8)	0.643
Remote infections	7 (3)	3 (1)	0.223
Non‐infectious postoperative complications	44 (17)	47 (19)	0.546
Non‐infectious CR‐POPF	6 (2)	13 (5)	0.078
Postoperative hospital stays	13 (IQR, 9–18)	17 (IQR, 12–26)	<0.001
Readmission	17 (7)	8 (3)	0.081
90‐day mortality	0	1 (0.4)	0.231

Abbreviations: ASA‐PS, American Society of Anesthesiologists physical status classification; BMI, body mass index; CD, Clavien–Dindo classification; CR‐POPF, clinically relevant postoperative pancreatic fistula; PNI, prognostic nutritional index; IQR, interquartile range.

With respect to postoperative outcomes, the intraoperative or up to 24‐h group had a significantly lower incidence of surgical site infections (not superficial incisional and deep incisional but organ/space infections, 17% vs. 29%, *p* = 0.001), infectious CR‐POPF (8% vs. 17%, *p* = 0.002), and prolonged postoperative hospital stay (13 vs. 17 days, *p* < 0.001). Severe surgical site infections were not significantly different between the two groups (*p* = 0.643). Other surgical outcomes, including remote infection, non‐infectious postoperative complications, non‐infectious CR‐POPF, readmission, and 90‐day mortality, were not significantly different between the two groups.

After PSM using the following nine variables — ASA‐PS, cardiovascular disease, pulmonary disease, smoking status, pancreatic disease, type of procedure, texture of the pancreas, method of pancreatic transection, and operation time — 181 patients were assigned to each group (Table 2). Patient characteristics were almost similar between the two groups. In terms of postoperative outcomes, the intraoperative or up to 24‐h group had a significantly lower incidence of surgical site infections (including superficial incisional and organ/space infections) at 19% compared with 33% in the more than 24‐h group (*p* = 0.003), as well as a lower rate of infectious CR‐POPF (8% vs. 17%, *p* = 0.006) and prolonged postoperative hospital stay (13 vs. 16 days, *p* < 0.001).

**TABLE 2:** Comparisons of patient characteristics and surgical outcomes between the intraoperative or up to 24‐h and more than 24‐h administration groups after propensity score matching (*n* = 362).

Factors	Antimicrobial prophylaxis		*p*
	Intraoperative or up to 24‐h, *n* = 181	More than 24‐h, *n* = 181	
Patient demographics			
Age, ≥80 years	27 (15)	25 (14)	0.764
Sex, female	100 (55)	96 (53)	0.673
BMI, ≥22 kg/m^2^	82 (45)	88 (49)	0.527
ASA‐PS, ≥3	15 (8)	18 (10)	0.584
Cardiovascular disease	20 (11)	17 (9)	0.603
Pulmonary disease	14 (8)	7 (4)	0.112
Diabetes	55 (30)	64 (35)	0.314
Laparotomy	51 (28)	62 (34)	0.212
Smoking	58 (32)	56 (31)	0.821
Steroids	4 (2)	1 (1)	0.162
PNI, <45	43 (24)	41 (23)	0.803
Pancreatic disease, malignant	102 (56)	91 (50)	0.246
Neoadjuvant therapy	50 (28)	63 (35)	0.140
Procedures, minimally invasive	85 (47)	83 (46)	0.833
Texture of pancreas, soft	166 (92)	162 (90)	0.471
Thickness of pancreas transection, ≥15 mm	53 (29)	52 (29)	0.908
Method of pancreas transection, stapler	169 (93)	168 (93)	0.836
Vascular resection	14 (8)	22 (12)	0.158
Organ resection	16 (9)	13 (7)	0.561
Operation time, ≥300 min	72 (40)	77 (43)	0.593
Estimated blood loss, ≥400 mL	41 (23)	48 (27)	0.393
Transfusion	9 (5)	10 (6)	0.814
Postoperative outcomes			
Surgical site infections	34 (19)	59 (33)	0.003
Superficial	2 (1)	9 (5)	0.026
Deep	1 (1)	1 (1)	1.000
Organ/space	31 (17)	53 (29)	0.006
Infectious CR‐POPF	14 (8)	31 (17)	0.006
CD ≥IIIa	16 (9)	16 (9)	1.000
Remote infections	5 (3)	2 (1)	0.245
Non‐infectious postoperative complications	28 (15)	34 (19)	0.402
Non‐infectious CR‐POPF	5 (3)	11 (6)	0.121
Postoperative hospital stays	13 (IQR, 9–17)	16 (IQR, 12–26)	<0.001
Readmission	12 (7)	7 (4)	0.236
90‐day mortality	0	1 (0.6)	0.239

Abbreviations: ASA‐PS, American Society of Anesthesiologists physical status classification; BMI, body mass index; CD, Clavien–Dindo classification; CR‐POPF, clinically relevant postoperative pancreatic fistula; PNI, prognostic nutritional index; IQR, interquartile range.

### 3.3 Risk factors of postoperative surgical site infection

Univariate and multivariate analyses of the risk factors for postoperative surgical site infections are summarized in Table 3. Univariate analysis showed that antimicrobial prophylaxis, thickness and method of pancreas transection, and operation time were significantly associated with surgical site infection. Multivariate logistic regression revealed that more than 24‐h administration of antimicrobial prophylaxis (OR = 2.04; *p* = 0.001), thicker pancreas transection (OR = 1.65; *p* = 0.023), non‐stapler transection of pancreas (OR = 2.13; *p* = 0.022), and longer operation time (OR = 1.92; *p* = 0.003) were independent risk factors for postoperative surgical site infections.

**TABLE 3:** Univariate and multivariate analysis of preoperative and surgical risk factors for surgical site infection (*n* = 496).

Factors	Univariate			Multivariate		
	Surgical site infection		*p*	OR	95% CI	*p*
	Yes, *n* = 129 (%)	No, *n* = 367 (%)				
Age, years						
<80	107 (83)	314 (86)	0.481			
≥80	22 (17)	53 (14)				
Sex						
Male	65 (50)	173 (47)	0.525			
Female	64 (50)	194 (53)				
BMI, kg/m^2^						
<22	58 (45)	200 (55)	0.062			
≥22	71 (55)	167 (45)				
ASA‐PS						
≤2	114 (88)	327 (89)	0.821			
≥3	15 (12)	40 (11)				
Cardiovascular disease						
Yes	20 (16)	44 (12)	0.314			
No	109 (84)	323 (88)				
Pulmonary disease						
Yes	16 (12)	40 (11)	0.645			
No	113 (88)	327 (89)				
Diabetes						
Yes	43 (33)	117 (32)	0.762			
No	86 (67)	250 (68)				
Laparotomy						
Yes	42 (33)	113 (31)	0.710			
No	87 (67)	254 (69)				
Smoking						
Yes	49 (38)	110 (30)	0.097			
No	80 (62)	257 (70)				
Steroids						
Yes	2 (2)	6 (2)	0.948			
No	127 (98)	361 (98)				
PNI						
<45	33 (26)	90 (25)	0.811			
≥45	96 (74)	277 (75)				
Pancreatic disease						
Borderline/benign	58 (45)	157 (43)	0.667			
Malignant	71 (55)	210 (57)				
Neoadjuvant therapy						
Yes	37 (29)	124 (34)	0.283			
No	92 (71)	243 (66)				
Procedures						
Minimally invasive	55 (43)	155 (42)	0.937			
Open	74 (57)	212 (58)				
Antimicrobial prophylaxis						
Intraoperative or up to 24 h	49 (38)	205 (56)	<0.001	1.0		0.001
More than 24 h	80 (62)	162 (44)		2.04	1.32–3.14	
Texture of pancreas						
Soft	105 (81)	318 (87)	0.156			
Firm	24 (19)	49 (13)				
Thickness of pancreas transection, mm						
<15	75 (58)	263 (72)	0.005	1.0		0.023
≥15	54 (42)	104 (28)		1.65	1.07–2.54	
Method of pancreas transection						
Stapler	109 (85)	338 (92)	0.017	1.0		0.022
Non‐stapler	20 (15)	29 (8)		2.13	1.11–4.06	
Vascular resection						
Yes	18 (14)	29 (8)	0.052			
No	111 (86)	338 (92)				
Organ resection						
Yes	13 (10)	35 (10)	0.859			
No	116 (90)	332 (90)				
Operation time, min						
<300	56 (43)	235 (64)	<0.001	1.0		0.003
≥300	73 (57)	132 (36)		1.92	1.26–2.93	
Estimated blood loss, mL						
<400	85 (66)	273 (74)	0.068			
≥400	44 (34)	94 (26)				
Transfusion						
Yes	10 (8)	21 (6)	0.423			
No	119 (92)	346 (94)				

Abbreviations: ASA‐PS, American Society of Anesthesiologists physical status classification; BMI, body mass index; CI, confidence interval; OR, odds ratio; PNI, prognostic nutritional index.

## 4 DISCUSSION

Antimicrobial prophylaxis is useful for the prevention of postoperative infections following gastrointestinal surgery. Its short‐term use is generally recommended,^1^, ^2^ as it minimizes the development of bacterial resistance, reduces the risk of antibiotic‐related complications, and is cheaper than prolonged regimens. However, specific evidence regarding the optimal duration of antimicrobial prophylaxis for each gastrointestinal surgical procedure remains controversial,^3^, ^4^, ^5^, ^6^ especially in pancreatic surgery.

Pancreatic surgery is a highly specific and difficult procedure, and postoperative complications including infectious diseases can occur even in high‐volume centers.^17^, ^18^, ^19^ Postoperative infections after pancreatic surgery primarily include surgical site infections (POPF, abdominal abscess, and anastomotic leakage) and remote infections (respiratory tract infections, antibiotic‐associated diarrhea, and urinary tract infections). Pancreatoduodenectomy includes pancreatojejunostomy/gastrostomy, hepaticojejunostomy, and gastro/duodenojejunostomy, and can cause POPF, bile leakage, anastomotic leakage, and various other infectious complications associated with gastrointestinal reconstruction. In addition, periampullary diseases with bile stenosis or obstructive jaundice require preoperative biliary drainage, and bile contamination is a risk factor for postoperative infection after pancreatoduodenectomy.^11^ Furthermore, the use of perioperative antibiotics covering bile contamination is valid for preventing postoperative infection. Antimicrobial prophylaxis and therapeutics may not be appropriate in most preoperative biliary drainage cases. In contrast, distal surgery does not require preoperative biliary drainage or intraoperative gastrointestinal anastomosis, and antimicrobial prophylaxis is relatively easy. In the current study, cephalosporins were used as antimicrobial prophylaxis for the eligible patients. Postoperative infection occurred in 28% of the eligible patients, including surgical site infection (26%) and remote infection (2%). Most surgical site infections were organ/space infections and almost half were infectious CR‐POPF. Nationwide surveys reported that postoperative infections after pancreatic surgery occurred in 15–31%^20^, ^21^, ^22^, ^23^ of patients, and our study results from four high‐volume centers were considered valid and reliable for the analysis of relationships between antimicrobial prophylaxis and postoperative infection after distal pancreatectomy. To the best of our knowledge, this is the first multicenter cohort study to report on the association between the duration of antimicrobial prophylaxis and postoperative infections after distal pancreatectomy. Moreover, the present study comprised a multi‐institutional cohort of patients who underwent surgery during the most recent 5‐year period, minimizing the bias of changes in usual practices, such as surgical procedures or treatment indications for postoperative infection.

Patients in this study were classified into two groups based on the duration of antimicrobial prophylaxis: intraoperative or up to 24‐h and more than 24‐h administration groups. Between the two groups, the more than 24‐h group had significantly worse performance status, cardiovascular and pulmonary disease, smoking, borderline tumors, soft pancreas, non‐stapler transection, and longer operation time. Some of them were previously reported as risk factors of postoperative infections.^24^, ^25^ These may represent an important bias in patient demographics in this retrospective study. With respect to postoperative outcomes, organ/space surgical site infections, mostly infectious CR‐POPF, occurred more frequently in the more than 24‐h group, whereas the frequencies of severe infections, remote infections, non‐infectious postoperative complications, readmission, and mortality were similar. These results were consistent in the PSM analysis and indicate that longer antimicrobial prophylaxis is highly associated with non‐severe infections. Clinically, whether longer antimicrobial prophylaxis causes infectious CR‐POPF or whether longer antimicrobial prophylaxis should be administered to high‐risk CR‐POPF patients remains controversial. However, multivariate logistic regression models revealed that more than 24‐h administration of antimicrobial prophylaxis was an independent risk factor for surgical site infections, as well as thicker pancreas transection, non‐stapler transection, and longer operation duration.

POPF is the most common postoperative complication following distal pancreatectomy. The definition and grading of POPF were developed and updated by an international study group.^8^ The risk factors for CR‐POPF include the following: small duct, soft pancreas, high‐risk pathology, and excessive blood loss.^26^ Longer antimicrobial prophylaxis was not included in the factors influencing fistula risk score. If prolonged antimicrobial prophylaxis causes infectious CR‐POPF, the underlying pathophysiological mechanism may involve antimicrobial resistance or microbial substitution. This longer administration could activate trypsin, elastase, and other pancreatic exocrine enzymes, leading to the development of infectious CR‐POPF, despite patients typically receiving supportive therapies, such as perioperative pancreatic enzymes, protectants, or early diets, to prevent these problems. In addition, prolonged antimicrobial prophylaxis may lead to occult bacterial translocation,^27^ a mild bacteremia not detected by conventional culture methods that can cause postoperative infections. In other surgical fields, prolonged prophylaxis with antimicrobials has also been associated with an increased risk of acquired antimicrobial resistance.^28^ Moreover, Krezalek et al.^29^ demonstrated that immune cells, such as those in Trojan horses, carrying gut‐derived antimicrobial‐resistant bacteria might be a plausible mechanism behind surgical site infections in the absence of direct contamination. Based on this theory, prolonged antimicrobial prophylaxis may be associated with postoperative infections.

This study has several limitations. First, patient selection, antimicrobial prophylaxis durations and protocols, surgical techniques, and approaches to postoperative infections may have varied among the centers, although PSM was conducted to minimize bias. However, four high‐volume centers in Japan were included in the study, all of which followed standard perioperative assessments in compliance with established guidelines. Second, it remains clinically unclear whether longer antimicrobial prophylaxis causes surgical site infections, or whether longer antimicrobial prophylaxis is administered to high‐risk surgical site infection patients. To overcome these limitations, further prospective randomized controlled trials with a larger number of institutions are warranted. In conclusion, prolonged administration of antimicrobial prophylaxis may not be effective in preventing surgical site infections after distal pancreatectomy compared to intraoperative or up to 24‐h administration.

## AUTHOR CONTRIBUTIONS

**Kenjiro Okada:** Conceptualization; data curation; formal analysis; investigation; methodology; project administration; writing – original draft; writing – review and editing. **Kenichiro Uemura:** Conceptualization; formal analysis; investigation; methodology; project administration; supervision; writing – review and editing. **Sohei Satoi:** Conceptualization; writing – review and editing. **Tsutomu Fujii:** Conceptualization; writing – review and editing. **Manabu Kawai:** Conceptualization; writing – review and editing. **So Yamaki:** Data curation; writing – review and editing. **Toru Watanabe:** Data curation; writing – review and editing. **Hideki Motobayashi:** Data curation; writing – review and editing. **Shinya Takahashi:** Conceptualization; formal analysis; investigation; methodology; project administration; supervision; writing – review and editing.

## FUNDING INFORMATION

The authors received no financial support for the research, authorship, and/or publication of this article.

## CONFLICT OF INTEREST STATEMENT

Author Tsutomu Fujii is an editorial board member of *Annals of Gastroenterological Surgery*.

## ETHICS STATEMENT

Approval of the research protocol by an Institutional Reviewer Board: The protocol for this research project has been approved by the Institutional Review Board of Hiroshima University, and it conforms to the provisions of the Declaration of Helsinki.

Informed Consent: N/A.

Registry and the Registration No. of the study/trial: Institutional Review Board of Hiroshima University, Approval No. E‐2022‐0227.

Animal Studies: N/A.
